# HIF1α Attenuated the Lung Ischemia–Reperfusion Injury by Activating the miR‐485/Notch1 Signalling

**DOI:** 10.1111/jcmm.70965

**Published:** 2026-01-06

**Authors:** Shaohua Dai, Xuemei Wan, Lingchun Xia, Lei Xu, Chunfan Xie, Guohui Wang, Jian Tang

**Affiliations:** ^1^ Department of Thoracic Surgery The First Affiliated Hospital of Nanchang University Nanchang China; ^2^ Department of Cardiovascular and Vascular Surgery The First Affiliated Hospital of Nanchang University Nanchang China

**Keywords:** hypoxia‐inducible factor 1‐alpha, lung ischemia–reperfusion injury, mitophagy, Notch1 signalling, pulmonary microvascular endothelial cells

## Abstract

Lung ischemia–reperfusion (I/R) injury is a common complication following lung transplantation and cardiac surgery. This study aimed to investigate the role and underlying mechanisms involving Neurogenic locus notch homologue protein 1 (Notch1) signalling and microRNA‐485 (miR‐485) of hypoxia‐inducible factor 1‐alpha (HIF1α) in protecting pulmonary microvascular endothelial cells (PMVECs) against I/R‐induced injury. We found that overexpression of HIF1α significantly increased cell viability and decreased apoptosis in hypoxia/reoxygenation (H/R)–treated PMVECs, while knockdown or inhibition of HIF1α had the opposite effects. Moreover, HIF1α overexpression activated the Notch1 signalling pathway by upregulating Hes1 mRNA expression and Hes1 promoter activity. Conversely, HIF1α knockdown or inhibition inhibited Notch1 signalling. Furthermore, HIF1α overexpression alleviated H/R‐induced mitophagy and mitochondrial dysfunction. On the other hand, HIF1α knockdown or inhibition aggravated mitophagy and mitochondrial dysfunction. We also found that HIF1α transcriptionally activated the expression of miR‐485. Overexpression of miR‐485 reversed the detrimental effects of HIF1α knockdown or inhibition on cell viability, apoptosis, Hes1 expression and mitophagy levels in H/R‐treated PMVECs. Additionally, we identified NUMB endocytic adaptor protein (Numb) mRNA as a direct target of miR‐485, and miR‐485 overexpression suppressed Numb expression in PMVECs. In conclusion, these results suggest that targeting the HIF1α‐miR‐485‐Numb axis may be a potential therapeutic strategy for attenuating lung I/R injury.

## 1 Introduction

Lung ischemia–reperfusion injury (IRI) refers to the damage that occurs when blood flow is temporarily interrupted and then restored to the lungs [1]. It is a common complication in various clinical scenarios, such as lung transplantation, cardiopulmonary bypass surgery and acute respiratory distress syndrome (ARDS) [2]. Understanding the research background and current status of lung IRI is crucial for developing effective therapeutic strategies.

Within this complex pathophysiological landscape, the interplay between specific molecular signalling pathways dictates the ultimate fate of the injured tissue. Two such critical and interconnected pathways are those mediated by Hypoxia‐Inducible Factor 1 Alpha (HIF1α) and the Notch signalling cascade. HIF1α is a master transcriptional regulator of the cellular adaptive response to hypoxia [3]. Under normoxic conditions, HIF1α is constitutively synthesised but rapidly degraded by the proteasome following prolyl hydroxylation and ubiquitination by the von Hippel–Lindau (VHL) E3 ligase complex [4]. During the ischemic phase of IRI, oxygen‐dependent hydroxylation is inhibited, leading to the rapid stabilisation and accumulation of the HIF1α protein [4]. This allows its translocation to the nucleus, heterodimerisation with HIF1β (ARNT) and binding to hypoxia‐response elements (HREs) to transactivate a vast array of genes crucial for survival under low oxygen tension [5]. These target genes are involved in diverse processes such as glycolysis (e.g., GLUT1, LDHA), angiogenesis (e.g., VEGF), erythropoiesis (EPO), apoptosis inhibition and inflammation modulation [6]. In the context of lung IRI, HIF1α activation has been shown to have a complex, context‐dependent role, but a growing body of evidence suggests its stabilisation can be profoundly protective by promoting metabolic adaptation and mitigating inflammatory and apoptotic signals.

Simultaneously, the evolutionarily conserved Notch signalling pathway is activated in response to the cellular stresses of IRI [7]. Notch signalling is initiated by the interaction between transmembrane Jagged or Delta‐like ligands on one cell and Notch receptors (Notch1‐4) on an adjacent cell [8]. This interaction triggers a series of proteolytic cleavages of the receptor, the most critical being mediated by the γ‐secretase complex, which releases the Notch Intracellular Domain (NICD) [9]. The NICD translocates to the nucleus where it associates with the transcription factor CSL (also known as RBP‐Jκ) and co‐activators of the Mastermind‐like (MAML) family. This complex then drives the expression of primary target genes, most notably the Hes (hairy and enhancer of split) and Hey (Hes‐related with YRPW motif) families of transcriptional repressors [10]. These effector genes exert wide‐ranging influences on cell fate decisions, proliferation, differentiation and inflammatory responses [11]. In lung IRI, the role of Notch signalling is equally nuanced and cell‐type specific. Evidence indicates that its activation can contribute to endothelial barrier disruption, promote pro‐inflammatory cytokine release and induce apoptosis [11]. Conversely, other studies demonstrate that specific inhibition of certain Notch receptors or ligands can attenuate injury, suggesting that the pathway's net effect is likely determined by the specific receptor‐ligand pairs engaged, the cellular context and the timing of activation [8].

The relationship between HIF1α and Notch signalling is not merely parallel but is deeply intertwined, forming a critical regulatory axis in the hypoxic response. A key mechanistic link exists, as HIF1α can transcriptionally upregulate the expression of Jagged1, a canonical Notch ligand, thereby potentiating Notch signalling activation in a hypoxic microenvironment [12]. Furthermore, a more direct molecular crosstalk has been established: the NICD can physically interact with HIF1α, inhibiting its VHL‐mediated ubiquitination and proteasomal degradation. This interaction effectively stabilises HIF1α protein even under normoxic conditions, dramatically amplifying the hypoxic transcriptional programme [13]. Reciprocally, HIF1α can bind to the NICD and enhance the transcription of Notch target genes, creating a potent positive feedback loop that integrates metabolic stress signals with developmental cell fate pathway [14]. This intricate cross‐talk positions the HIF1α‐Notch axis as a central coordinator of the cellular response to I/R stress, yet the precise mechanisms and downstream effectors through which this axis confers protection or exacerbates damage in lung microvasculature remain incompletely elucidated.

A crucial point of regulation within the Notch pathway is the protein Numb [15]. Numb is an evolutionarily conserved adaptor protein that functions as a potent cell‐fate determinant and a key intrinsic negative regulator of Notch signalling [16]. It achieves this by promoting the endocytic degradation of Notch receptors and their ligands, thereby finely tuning the amplitude and duration of Notch activation [17, 18]. The functional antagonism between Numb and Notch is fundamental to numerous asymmetric cell divisions and developmental processes [19]. In the context of injury and stress, the expression level of Numb can therefore act as a molecular switch, either permitting or restraining Notch‐mediated responses [20]. Consequently, understanding the upstream regulation of Numb is critical to mapping the control network of the Notch pathway during IRI.

Emerging as pivotal upstream regulators of gene expression are microRNAs (miRNAs), small non‐coding RNAs that post‐transcriptionally silence target genes by binding to complementary sequences in the 3′ untranslated regions (3′ UTRs) of mRNAs, leading to their degradation or translational repression [21]. One such miRNA, miR‐485, has been implicated in various cellular processes, including neuronal development [22, 23, 24]. Intriguingly, bioinformatic analyses predict that the 3′ UTR of Numb mRNA contains highly conserved binding sites for miR‐485, suggesting a direct regulatory relationship wherein miR‐485 could suppress Numb expression. This potential interaction places miR‐485 in a strategic position to influence the Notch signalling pathway indirectly by modulating the levels of its key negative regulator, Numb. The transcriptional control of miR‐485 itself, particularly under hypoxic conditions, remains an open area of investigation, with potential links to known hypoxia‐responsive transcription factors like HIF1α being a compelling possibility.

In this study, we found that HIF1α protects pulmonary microvascular endothelial cells (PMVECs) against I/R‐induced injury and mitophagy by activating the Notch1 signalling pathway and transcriptionally upregulating miR‐485 expression. Furthermore, miR‐485 targets Numb mRNA and inhibits its expression, thus contributing to the protective effects of HIF1α. These results suggest that targeting the HIF1α‐miR‐485‐Numb axis may be a potential therapeutic strategy for attenuating lung I/R injury.

## 2 Materials and Methods

Approval for the study protocols and animal experimentation was granted by the Animal Ethics Committee of the First Affiliated Hospital of Nanchang University. Significant efforts were made to reduce the utilisation and distress of the animals involved in the research.

### 2.1 PMVEC Cell Culture and H/R Injury Model

PMVECs were acquired from Cell Biologics Inc., located in Chicago, IL, USA. In vitro, the cell models for H/R injury were established using a technique outlined in prior publications [25, 26, 27]. In short, mouse PMVECs were suspended in M‐1168 (Cell Biologics, Chicago, IL) with supplements, including 10% FBS, 1% Penicillin and Streptomycin (Gibco) and incubated at 37°C in a 5% CO_2_–95% air environment. The passage range (3–8) was used for PMVECs. Based on the rate of cell growth, the density of cells was modified and then they were placed in 6‐well plates. When the cells reached 80%–90% confluence, they were transfected with the Lipofectamine 2000 reagent (Invitrogen, Carlsbad, CA). Following H/R treatment, the cells were then transfected with a plasmid for over‐expressing HIF1α, HIF1α siRNA, mimic/inhibitor NC and miR‐485 mimic/inhibitor (50 nM). Following a 6‐h transfection period, the medium was substituted, and subsequently, the cells were cultivated for an additional 24 h. Afterwards, the cells were incubated in a low‐oxygen environment with 95% nitrogen, 4% carbon dioxide and 1% oxygen for 12 h. Subsequently, they were transferred to a regular cell incubator and cultured for an additional 4 h to establish a H/R cell model. Following this, cellular samples were gathered from every group for further experimentation.

### 2.2 Cells Viability Assay

The viability of PMVECs was determined by utilising the CCK‐8 kit from Shanghai Beyotime Biotechnology Co. Ltd., Shanghai, China, in accordance with the guidelines provided by the manufacturer. Under hypoxic conditions, cells were plated on 96‐well plates with a density of 1 × 10^4^ cells per well (100 μL/well) and subjected to H/R and Propofol treatment. Propofol was administered multiple times in six wells at a concentration of 100 μm/L. Non‐H/R cells were treated with Propofol for 16 h. Following treatment, the cells were washed twice with PBS and then incubated with 10 μL of CCK8 solution at 37°C for 2 h. A microplate reader was used to determine the absorbance value at 450 nm. Cell viability (%) was calculated using the formula [(As−Ab)/(Ac−Ab)] × 100%, where ‘As’ represents the absorbance value of supernatant from exposed or sham exposure dishes, ‘Ac’ represents the absorbance value of pore containing supernatant in normal control, and ‘Ab’ represents the absorbance value of cultured pore containing 10% CCK8 solution.

### 2.3 Flow Cytometry

The detection of cell apoptosis was performed using the apoptosis detection kit (Shanghai Beyotime Biotechnology Co. Ltd., Shanghai, China) with Annexin V‐fluorescein isothiocyanate (FITC) and propidium iodide (PI). After being washed twice with cold PBS, a million cells per millilitre were gently resuspended with 195 μL of Annexin V‐FITC binding solution. Afterward, cells were cultured with 5 μL Annexin V‐FITC and 10 μL PI at ambient temperature in the absence of light for a duration of 15 min. The quantification of cell apoptosis was performed using a flow cytometer (FACSVerse/Calibur/AriaIISORP, BD Biosciences, San Jose, CA, USA).

### 2.4 Real‐Time qPCR

The TRIzol reagent was used to extract the total RNA content from both lung tissues and cells. Using a Nano‐Drop 2000 spectrophotometer (Thermo Fisher Scientific, Rockford, IL), the RNA concentration was measured. To reverse transcribe the RNA into cDNA, we utilised the ReverTra Ace qPCR RT Master Mix with gDNA remover kit from Toyobo Co. Ltd. in Osaka, Japan. Every inquiry consisted of a minimum of three wells, with each well being replicated three times. Sangon Biotech Co. Ltd. (Shanghai, China) synthesised the primers for HIF1α, Notch1, Hes1, Numb and miR‐484. U6 and glyceraldehyde‐3‐phosphate dehydrogenase were used as internal references. Afterward, RT‐qPCR was conducted following the guidelines of the 2× Real‐Star Green Fast Mixture Kit (Genstar, China). The fold changes were determined using the method of relative quantification, specifically the 2^−ΔΔCt^ approach.

### 2.5 Mitophagy Assay by Immunofluorescence Staining

PMVECs were treated with Mito‐Tracker Red CMXRos from Shanghai Beyotime Biotechnology Co. Ltd., Shanghai, China for 30 min. After that, they were fixed using 4% paraformaldehyde and permeabilised with 0.3% Triton X‐100. Bovine serum albumin (BSA) was applied to block the cells. Next, the cells were incubated overnight at 4°C with rabbit anti‐LC3B antibody (ab51520, 1:2000, Abcam). Finally, the cells were incubated for another hour at ambient temperature with Alexa Fluor 488‐labelled donkey anti‐rabbit IgG secondary antibody (A21206, 1:500, Thermo Fisher Scientific, Waltham, MA, USA). Subsequently, we employed the confocal laser microscope (LSM800, Zeiss, Germany) for cell observation. A total of 200 or more cells were computed from each section to establish the ratio of MitoTracker Red and LC3 spot cells (mitophagy).

### 2.6 Western Blot Analysis

Cells from each group (OE‐NC, OE‐HIF1α, si‐NC, si‐HIF1α, Blank, Genistein, miR‐NC, miR‐485 mimic, Inhibitor‐NC, miR‐485 inhibitor) were collected and lysed on ice for 5 min using radio‐immunoprecipitation assay lysis buffer (provided by Shanghai Beyotime Biotechnology Co. Ltd., Shanghai, China). The supernatant was obtained after being subjected to centrifugation at a speed of 12,000 rpm and a temperature of 4°C. Next, the bicinchoninic acid (Pierce, Thermo Fisher Scientific, Waltham, MA) method was employed to ascertain the protein concentration. The protein was isolated by employing a 4% separation gel and a 10% spacer gel and subsequently transferred onto a membrane made of polyvinylidene fluoride. Afterward, the membrane underwent treatment with 5% skim milk powder at room temperature for 1 h. It was then incubated overnight at 4°C with primary antibodies against HIF1A (dilution ratio of 1:2000), Notch1 (dilution ratio of 1:1000), Hes1 (dilution ratio of 1:100), p‐MFN1 (dilution ratio of 1:1500), MFN1 (dilution ratio of 1:1500), p‐DRP1 (dilution ratio of 1:2000) and DRP1 (dilution ratio of 1:3000) (all obtained from Abcam Inc., Cambridge, MA, USA). After the incubation period, the membrane underwent retesting using a secondary antibody IgG (Santa Cruz Biotechnology Inc., Santa Cruz, CA) labelled with horseradish peroxidase. This step was carried out at room temperature for 1 h following a wash with Tris‐buffered saline Tween‐20, also at room temperature. Subsequently, the membrane was observed utilising an enhanced chemiluminescence reaction provided by Thermo Fisher Scientific located in Rockford, IL. The protein bands' volume was measured using a Bio‐Rad ChemiDoc EQ densitometer and the Bio‐Rad Quantity One v4.6.2 software. The protein expression was indicated by the ratio of the target band's grey value to that of glyceraldehyde‐3‐phosphate dehydrogenase (Abcam Inc., Cambridge, MA).

### 2.7 Dual‐Luciferase Reporter Assay

The luciferase report vectors (Genelily Biotechnology Co. Ltd., Shanghai, China) were used to introduce the predicted fragments of Numb with miR‐485, including the 3′‐untranslated region (UTR), binding site and mutation fragments, as report plasmids Numb–wild‐type (WT) and Numb–mutant. Next, Numb mRNA luciferase report plasmids were co‐transfected with NC and miR‐485 mimic to investigate the potential binding between Numb and miR‐485. Following a 48‐h period of transfection, the cells were gathered and subjected to lysis. The following steps were conducted utilising a luciferase assay kit (K801‐200, Biovision, Bay Area, San Francisco, CA) with the dual‐luciferase reporter gene analysis system (Promega Corporation, Madison, WI).

### 2.8 Mouse Model Establishment of Lung I/R Injury in Orthotopic Mouse Lung Transplants

The models of lung injury caused by ischemia/reperfusion (I/R) were created following the previously explained methods. Shanghai SLAC Laboratory Animal Co. Ltd. (Shanghai, China) provided 60 male C57 mice, aged 6–8 weeks and weighing 21–26 g, which were specific pathogen‐free. The obtained mice were kept in an environment with alternating periods of 12 h of light and darkness and were utilised to create mouse models of lung IRI. The transplantation procedure was performed as described before [28, 29]. The cold ischaemic time was 2 h. The transplanted graft and the right, non‐transplanted lung were analysed at two time points, at 30 min and 4 h after reperfusion.

### 2.9 Haematoxylin–Eosin Staining

Lung tissues were fixed, dehydrated, paraffin‐embedded and sliced into 5‐μm‐thick sections. Next, the sections were placed on a prepared slide, which was then put in a 60°C incubator overnight. The sections were subsequently dewaxed, immersed in xylene I for 20 min, xylene II for 20 min, rehydrated with gradient alcohol (100%, 95%, 80% and 70%, 5 min each), rinsed with 0.01 M PBS 3 times (5 min each), and stained with haematoxylin for 2–3 min (nuclei), followed by 3 rinses with PBS (5 min each). Thereafter, the sections were immersed in 1% hydrochloric acid‐ethanol for 2–3 s, rinsed with PBS 3 times (5 min each), stained with eosin for 1 min, and then rinsed again with PBS 3 times (5 min each). Afterward, the sections were dehydrated with alcohol (70%, 80%, 95% and 100%, 5 min each), cleared with xylene for 10 min, dried and sealed using neutral gum. Two uninformed investigators performed the histological analysis under a microscope according to the standard of Lung Injury Score, and the final score was determined by the average value of the two investigators. The Lung Injury Score consists of grades (0–4), with 0 being normal and 4 indicating severe injury, including alveolar hyperemia, haemorrhage, interstitial edema, neutrophil count and alveolar wall thickness.

### 2.10 Detection of Lung Wet Weight‐To‐Dry Weight Ratio

At first, one out of every three tissues from the left lung was selected, and its weight was measured using an OHAUS‐Precision electronic balance (wet weight) before being recorded. Afterward, the tissues were placed inside an oven set at a temperature of 60°C for a duration of 72 h until reaching a stable weight, after which the dry weight was measured. Finally, the wet weight‐to‐dry weight (W/D) ratio was computed to indicate the swelling of the lung tissues.

### 2.11 Statistical Analysis

The SPSS 21.0 statistical software (IBM Corp. Armonk, NY) was utilised for processing all the data. The measurement data were represented as the average plus or minus the standard deviation. When the data followed a normal distribution and had homogeneity of variance, the comparisons between two unpaired groups were analysed using an unpaired *t*‐test. The analysis involved comparing multiple groups using 1‐way ANOVA along with Tukey's post hoc test. The correlation coefficient of Pearson was utilised to examine the connection among the indicators. A significance level of *p* < 0.05 was deemed statistically significant.

## 3 Results

### 3.1 HIF1α Alleviated H/R Induced PMVECs Injury and Activating Notch1 Signalling Pathway

**FIGURE 1:**
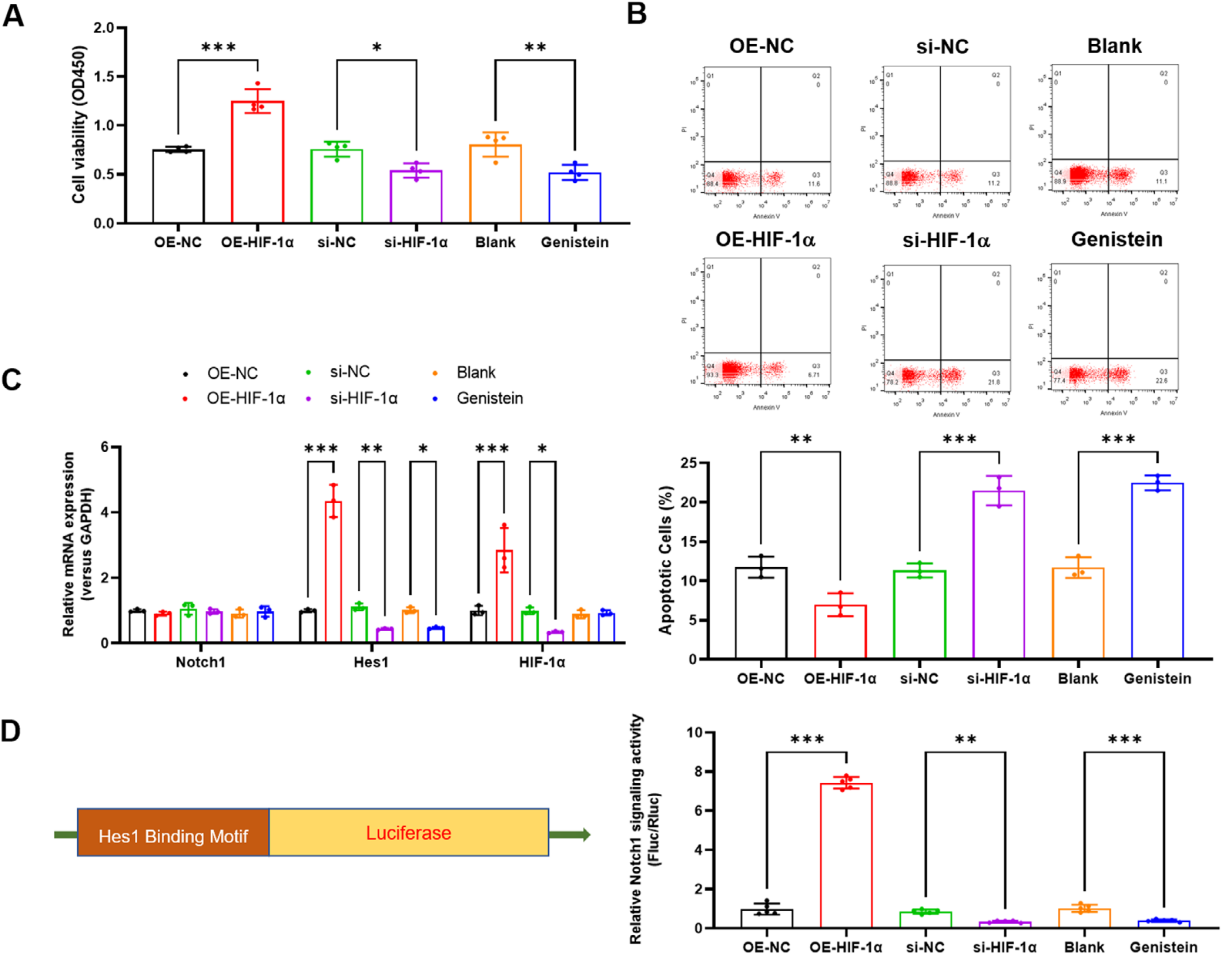
HIF1α alleviated H/R‐induced PMVECs injury and activated the Notch1 signalling pathway. (A) The effect of HIF1α overexpression, knockdown or inhibition on PMVECs cell viability was assessed by CCK‐8 assay; (B) The effect of HIF1α overexpression, knockdown or inhibition on PMVECs apoptosis was assessed by flow cytometry; (C) The effect of HIF1α overexpression, knockdown or inhibition on the expression of Notch1, Hes1 and HIF1α was analysed by real‐time PCR; (D) The effect of HIF1α overexpression, knockdown or inhibition on the promoter activity of Hes1 was analysed by luciferase assay. *N* = 3, *p < 0.05, **p < 0.01, ***p < 0.001, compared with the indicated group. H/R, hypoxia/reoxygenation; Hes1, Hes family BHLH transcription factor 1; HIF1α, hypoxia‐inducible factor 1‐alpha; Notch1, neurogenic locus notch homologue protein 1; PMVECs, pulmonary microvascular endothelial cells.

To understand the effect of HIF1α in Lung I/R Injury and the Notch1 signalling pathway, we overexpressed or knocked down the level of HIF1α in PMVECs. Subsequently, H/R PMVECs cell models were constructed, followed by relevant in vitro experimentation. HIF1α overexpression significantly increased the cell viability of PMVECs (*p* < 0.001), whereas HIF1α knockdown decreased it in PMVECs (*p* = 0.03), akin to the effect of Genistein (HIF1α inhibitor) (Figure 1A). HIF1α overexpression also significantly decreased the H/R‐induced apoptosis of PMVECs (*p* = 0.009), while HIF1α knockdown and inhibition increased the H/R‐induced apoptosis in PMVECs (*p* < 0.001) (Figure 1B). Moreover, HIF1α overexpression dramatically increased the expression level of Hes1 mRNA (*p* < 0.001), whereas it did not affect the expression level of Notch1. In contrast, HIF1α knockdown (*p* = 0.007) and inhibition (*p* = 0.04) similarly decreased the expression level of Hes1 mRNA (Figure 1C). Consistently, HIF1α overexpression dramatically increased the promoter activity of Hes1 (*p* < 0.001), whereas HIF1α knockdown (*p* = 0.003) and inhibition (*p* < 0.001) similarly decreased it (Figure 1D). These results suggested that HIF1α alleviated H/R‐induced PMVECs injury and activated the Notch1 signalling pathway.

### 3.2 HIF1α Alleviated H/R‐Induced Mitophagy and Mitochondrial Dysfunction

**FIGURE 2:**
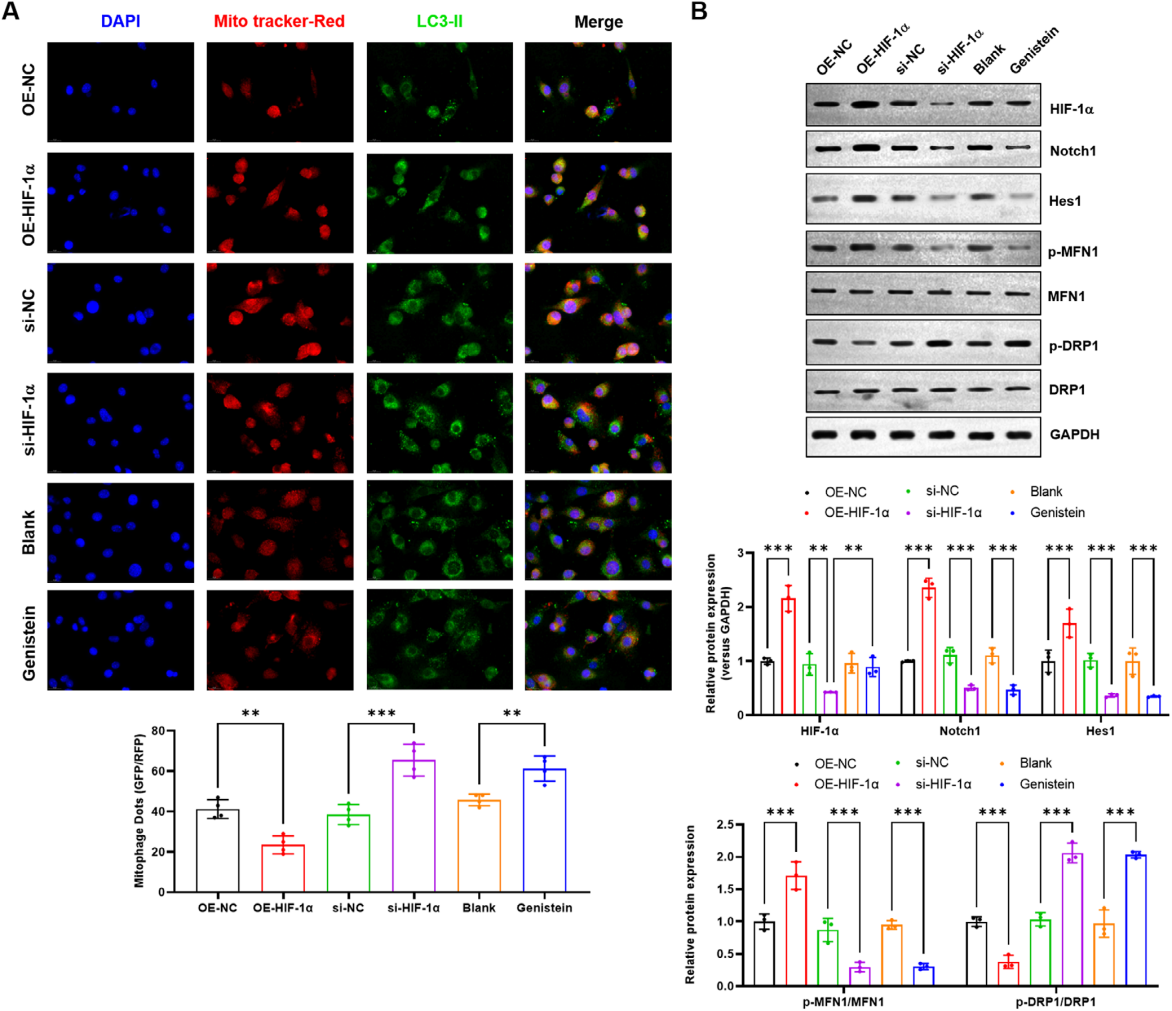
HIF1α alleviated H/R‐induced mitophagy and mitochondrial dysfunction. (A) The effect of HIF1α overexpression, knockdown or inhibition on mitophagy was analysed by IF assay; (B) The effect of HIF1α overexpression, knockdown or inhibition on the protein levels of HIF1α, Notch1, Hes1, p‐MFN1, MFN1, p‐DRP1 and DRP1 was analysed by Western blotting. *N* = 3, **p < 0.01, ***p < 0.001, compared with the indicated group. DRP1, dynamin‐related protein 1; H/R, hypoxia/reoxygenation; Hes1, Hes family BHLH transcription factor 1; HIF1α, hypoxia‐inducible factor 1‐alpha; IF, immunofluorescence; MFN, mitofusin‐1; Notch1, neurogenic locus notch homologue protein 1.

We previously found that Notch1 signalling inhibits mitophagy and mitochondrial dysfunction in lung I/R injury [30]. Thus, we analysed the mitophagy and mitochondrial dysfunction in the HIF1α overexpression, knockdown or inhibition PMVECs. As shown in Figure 2A, the mitophagy levels were obviously inhibited by HIF1α (*p* = 0.002), indicated by the Mito Tracker‐Red and LC3II positive dots. HIF1α knockdown (*p* < 0.001) and inhibition (*p* = 0.002) significantly increased the H/R‐induced mitophagy levels in PMVECs (Figure 2A). HIF1α over‐expression also increased the protein level of Notch1 and Hes1 (Figure 2B, *p* < 0.001). In contrast, HIF1α knockdown and inhibition decreased the protein level of Notch1 and Hes1 (Figure 2B, *p* < 0.001). The mitochondrial dysfunction was evaluated by the levels of mitochondrial fusion MFN1 and fission marker DRP1. HIF1α over‐expression significantly increased the protein level of phosphorylated MFN1 and decreased the phosphorylated DRP1 (Figure 2B, *p* < 0.001), suggesting an alleviated mitochondrial dysfunction. In contrast, HIF1α knockdown and inhibition increased the protein level of phosphorylated MFN1 and increased the phosphorylated DRP1, suggesting an aggravating mitochondrial dysfunction (Figure 2B, *p* < 0.001). These results showed that HIF1α alleviated H/R‐induced mitophagy and mitochondrial dysfunction.

### 3.3 HIF1α Transcriptionally Activates the Expression of miR‐485

**FIGURE 3:**
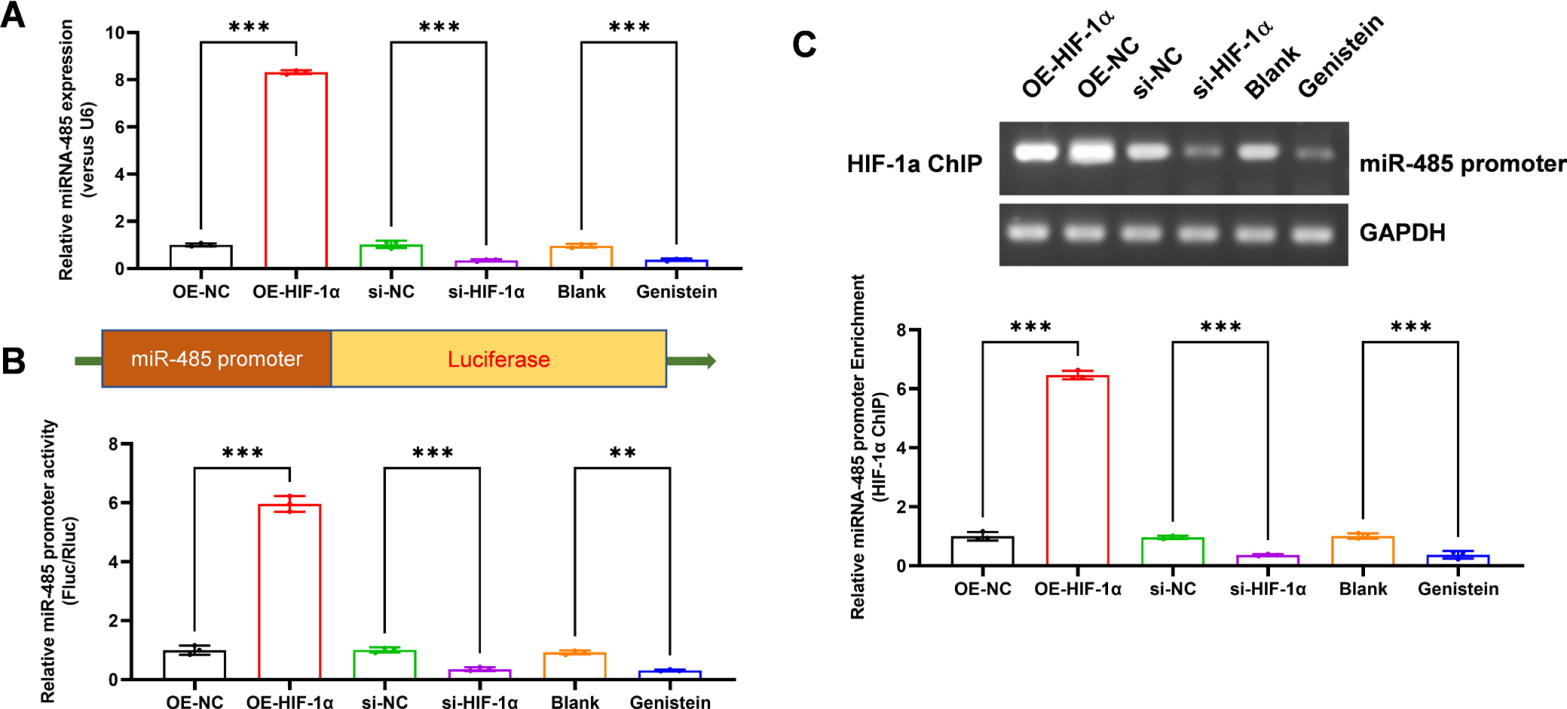
HIF1α transcriptionally activates the expression of miR‐485. (A) Effect of HIF1α overexpression, knockdown or inhibition on the expression of miR‐485 was analysed by real‐time PCR; (B) Effect of HIF1α overexpression, knockdown or inhibition on the promoter activity of miR‐485 was analysed by luciferase assay; (C) Effect of HIF1α overexpression, knockdown or inhibition on the interaction between HIF1α and miR‐485 promoter was analysed by ChIP assay. **p < 0.01, ***p < 0.001, compared with indicated group. *N* = 3, ChIP, chromatin immunoprecipitation; HIF1α, hypoxia‐inducible factor 1‐alpha.

Previous studies showed that HIF1α could target the promoter region of miR‐485 and regulate its expression. We thus evaluated the expression level of miR‐485 in the HIF1α overexpression, knockdown or inhibition of H/R PMVECs. Interestingly, HIF1α overexpression significantly increased the expression level of miR‐485, whereas HIF1α knockdown and inhibition decreased it in PMVECs (Figure 3A, *p* < 0.001). Furthermore, we constructed a miR‐485 promoter activity reporter with luciferase, which showed that HIF1α overexpression significantly increased the expression level of miR‐485 (*p* < 0.001), whereas HIF1α knockdown (*p* < 0.001) and inhibition (*p* = 0.001) decreased it in PMVECs (Figure 3B). HIF1α ChIP assay also demonstrated that HIF1α overexpression enhanced the interaction between HIF1α and the miR‐485 promoter, which was suppressed by HIF1α knockdown or inhibition (Figure 3C, *p* < 0.001). These results demonstrated that HIF1α transcriptionally activates the expression of miR‐485.

### 3.4 HIF1α Alleviated H/R Induced PMVECs Injury and Mitophagy via miR‐485

**FIGURE 4:**
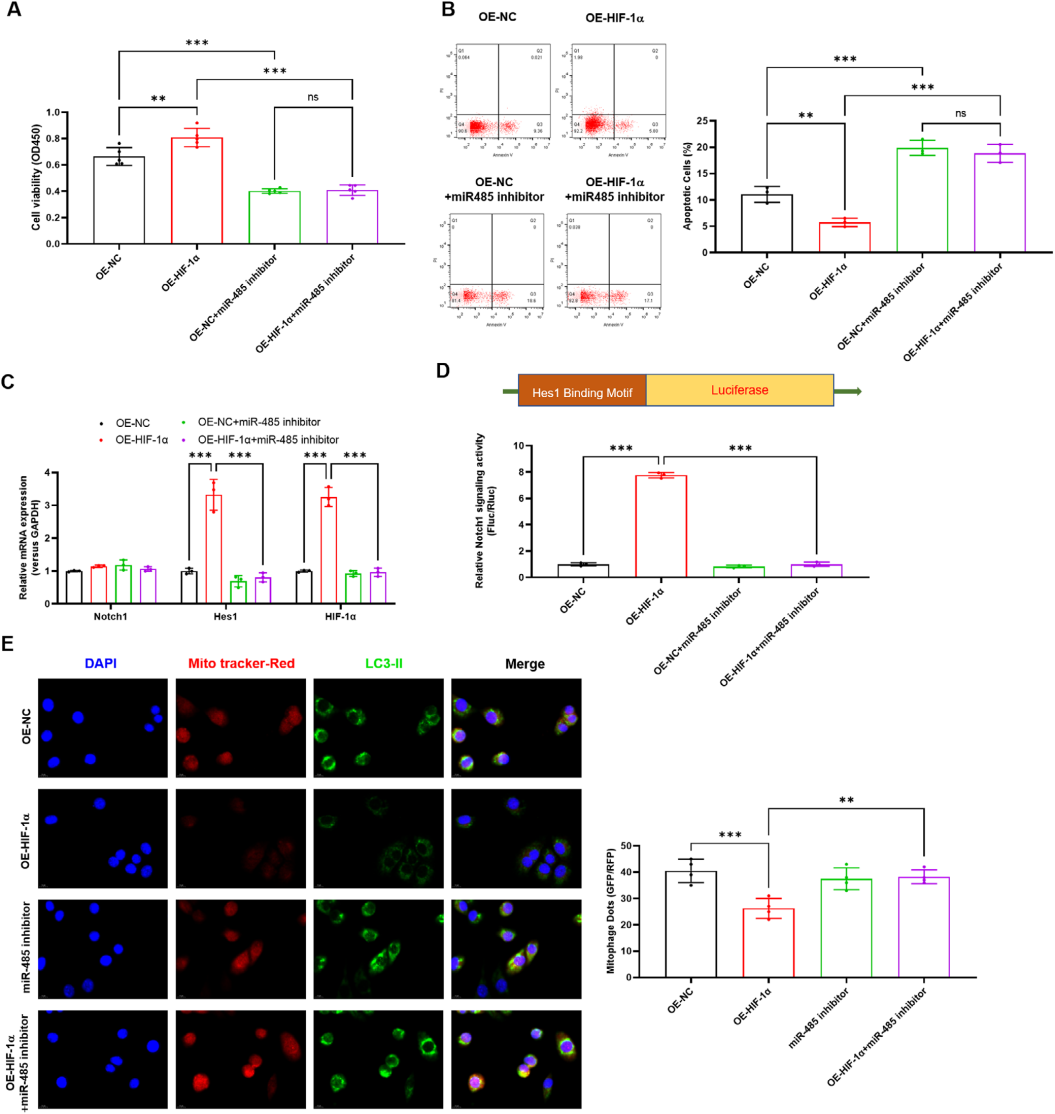
HIF1α alleviated H/R induced PMVECs injury and mitophagy via miR‐485. (A) Effect of HIF1α overexpression and miR‐485 inhibition on PMVECs cell viability was assessed by CCK‐8 assay; (B) Effect of HIF1α overexpression and miR‐485 inhibitor on PMVECs apoptosis was assessed by flow cytometry; (C) Effect of HIF1α overexpression and miR‐485 inhibitor on the expression of Notch1, Hes1 and HIF1α was analysed by real‐time PCR; (D) Effect of HIF1α overexpression and miR‐485 inhibitor on the promoter activity of Hes1 was analysed by luciferase assay; (E) Effect of HIF1α overexpression and miR‐485 inhibitor on mitophagy was analysed by IF assay. **p < 0.01, ***p < 0.001, compared with indicated group. *N* = 3, H/R, hypoxia/reoxygenation; Hes1, Hes family BHLH transcription factor 1; HIF1α, hypoxia‐inducible factor 1‐alpha; IF, immunofluorescence; Notch1, neurogenic locus notch homologue protein 1; PMVECs, pulmonary microvascular endothelial cells.

To further explore the role of miR‐485 in the HIF1α alleviated H/R‐induced PMVECs injury and mitophagy. We transfected the miR‐485 in vector control and HIF1α overexpression PMVECs and underwent H/R treatment. It was shown that HIF1α overexpression obviously increased the cell viability of H/R PMVECs (*p* = 0.003), which was suppressed (*p* < 0.001) by miR‐485 inhibitor transfection (Figure 4A). Similarly, the HIF1α decreased (*p* = 0.007) apoptosis of H/R PMVECs was elevated (*p* < 0.001) by miR‐485 inhibitor transfection (Figure 4B). Moreover, the HIF1α elevated level of Hes1 mRNA expression (Figure 4C, *p* < 0.001) and Hes1 promoter activity (Figure 4D, *p* < 0.001) were both suppressed by miR‐485 inhibitor transfection (*p* < 0.001). As shown in Figure 4E, the mitophagy levels were obviously inhibited by HIF1α (*p* < 0.001), indicated by the Mito Tracker‐Red and LC3II positive dots, which were obviously increased by miR‐485 inhibitor transfection in H/R PMVECs (*p* = 0.004). These results revealed that HIF1α alleviated H/R‐induced PMVECs injury and mitophagy via miR‐485.

### 3.5 miR‐485 Targets the Numb mRNA and Inhibits Its Expression in PMVECs

**FIGURE 5:**
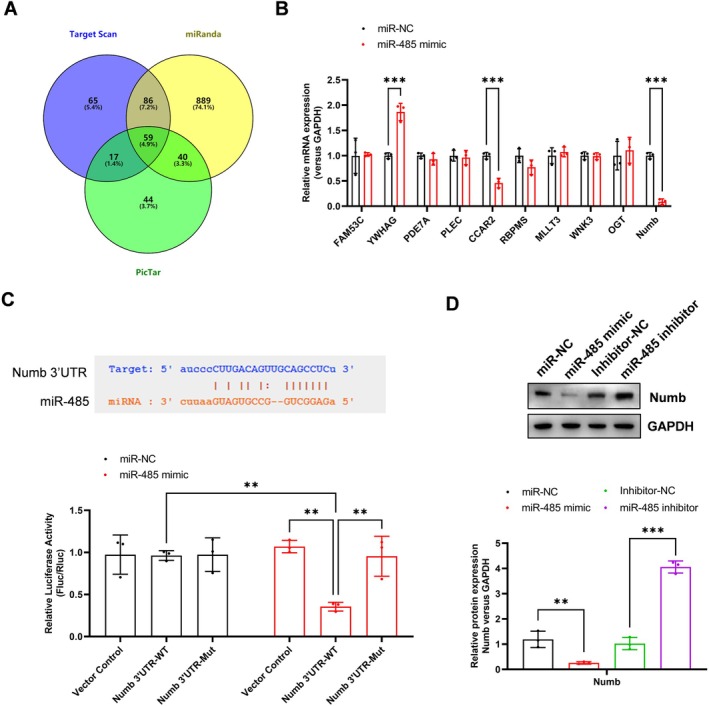
miR‐485 targets the Numb mRNA and inhibits its expression in PMVECs. (A) The potential targets of miR‐485 were predicted with three miRNAs target prediction tools, including Target Scan, miRanda and Picta; (B) The top 10 potential targets were verified by real‐time qPCR; (C) The dual‐luciferase assay confirmed that miR‐485 targeted the 3′ UTR region of Numb mRNA; (D) Western blotting analysis verified that the miR‐485 mimic inhibited the protein level of Numb, whereas the miR‐485 inhibitor increased the protein level of Numb. **p < 0.01, ***p < 0.001, compared with the indicated group. *N* = 3, 3′ UTR, 3′ untranslated regions; HIF1α, hypoxia‐inducible factor 1‐alpha; Numb, NUMB endocytic adaptor protein; PMVECs, pulmonary microvascular endothelial cells.

To understand the regulation mechanism of HIF1α‐miR‐485 in H/R‐induced PMVECs injury and mitophagy, we screened the potential targets of miR‐485 with three miRNA target prediction tools, including Target Scan, miRanda and Pictar (Figure 5A, *p* < 0.001). The top 10 potential targets were verified by real‐time qPCR, which showed that YWHAG, CCAR2 and Numb were significantly inhibited by miR‐485 mimic transfection (Figure 5B, *p* < 0.001). Due to the negative regulator of Notch signalling, we selected Numb for further validation. The dual‐luciferase assay confirmed that miR‐485 targeted the 3′ UTR region of Numb mRNA and suppressed its expression (Figure 5C, *p* < 0.001). Western blotting analysis further verified that the miR‐485 mimic inhibited the protein level of Numb (*p* = 0.007), whereas the miR‐485 inhibitor increased the protein level of Numb (Figure 5D, *p* < 0.001). These results showed that miR‐485 targets the Numb mRNA and inhibits its expression in PMVECs.

### 3.6 HIF1α Alleviated H/R Induced PMVECs Injury and Mitophagy via miR‐485 Suppressed Numb Expression

**FIGURE 6:**
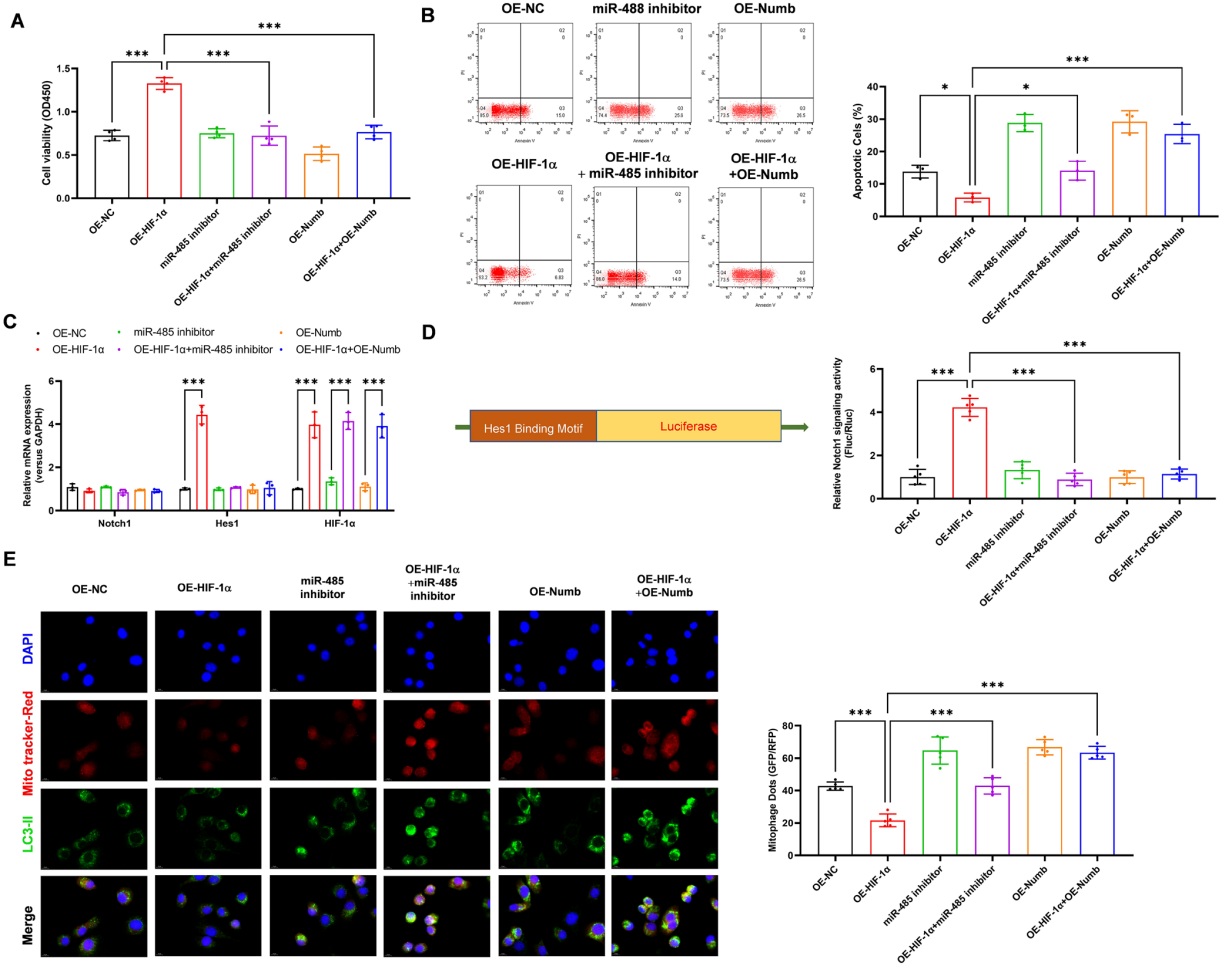
HIF1α alleviated H/R induced PMVECs injury and mitophagy via miR‐485 suppressed Numb expression. (A) Effect of HIF1α overexpression with miR‐485 inhibition or Numb overexpression on PMVECs cell viability was assessed by CCK‐8 assay; (B) Effect of HIF1α overexpression with miR‐485 inhibition or Numb overexpression on PMVECs apoptosis was assessed by flow cytometry; (C) Effect of HIF1α overexpression with miR‐485 inhibition or Numb overexpression on the expression of Notch1, Hes1 and HIF1α was analysed by real‐time PCR; (D) Effect of HIF1α overexpression with miR‐485 inhibition or Numb overexpression on the promoter activity of Hes1 was analysed by luciferase assay; (E) Effect of HIF1α overexpression with miR‐485 inhibition or Numb overexpression on mitophagy was analysed by IF assay. *p < 0.05, ***p < 0.001, compared with the indicated group. *N* = 3, H/R, hypoxia/reoxygenation; Hes1, Hes family BHLH transcription factor 1; HIF1α, hypoxia‐inducible factor 1‐alpha; IF, immunofluorescence; Notch1, neurogenic locus notch homologue protein 1; Numb, NUMB endocytic adaptor protein; PMVECs, pulmonary microvascular endothelial cells.

To further verify the critical role of miR‐485 in suppressed Numb expression in HIF1α alleviated H/R induced PMVECs injury and mitophagy, we transfected the miR‐485 inhibitor or Numb overexpression in vector control or HIF1α overexpression H/R PMVECs. It was shown that HIF1α overexpression obviously increased the cell viability of H/R PMVECs, which was suppressed by miR‐485 inhibitor or Numb overexpression transfection (Figure 6A, *p* < 0.001). Similarly, the HIF1α decreased apoptosis (*p* = 0.03) of H/R PMVECs was elevated by miR‐485 inhibitor (*p* = 0.02) or Numb overexpression (*p* < 0.001) transfection (Figure 6B). Moreover, the HIF1α elevated level of Hes1 mRNA expression (Figure 6C, *p* < 0.001) and Hes1 promoter activity (Figure 6D, *p* < 0.001) were both suppressed by miR‐485 inhibitor or Numb overexpression transfection. As shown in Figure 6E, the mitophagy levels were obviously inhibited by HIF1α (*p* < 0.001), indicated by the Mito Tracker‐Red and LC3II positive dots, which were obviously increased by miR‐485 inhibitor (*p* < 0.001) or Numb overexpression transfection (*p* < 0.001) in H/R PMVECs. These results revealed that HIF1α alleviated H/R induced PMVECs injury and mitophagy via miR‐485 suppressed Numb expression.

### 3.7 HIF1α Alleviated I/R‐Induced Lung Injury and Mitophagy via miR‐485 Suppressed Numb Expression In Vivo

**FIGURE 7:**
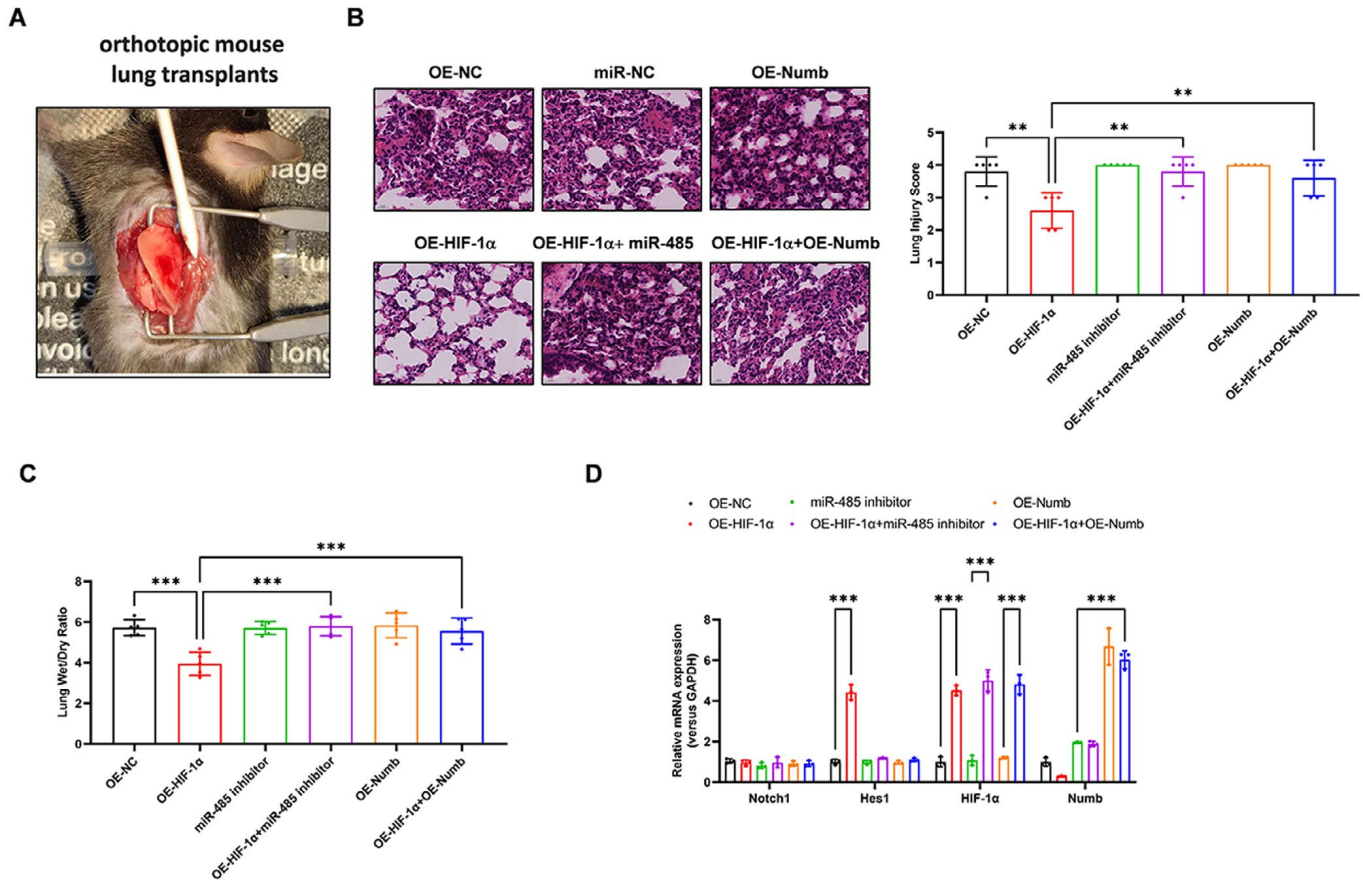
HIF1α alleviated I/R‐induced lung injury and mitophagy via miR‐485 suppressed Numb expression in vivo. (A) Mouse models with lung I/R injury in orthotopic mouse lung transplants were established; (B) HE staining analysis (×400) of mouse lung tissues and Lung Injury Score; (C) W/D ratio in mouse lung tissues was weighed with an electronic balance; (D) The level of Notch1, Hes1, HIF1α and Numb expression in lung tissues was analysed by real‐time PCR. *N* = 6, **p < 0.01, ***p < 0.001, compared with indicated group. HE, haematoxylin–eosin staining; Hes1, Hes family BHLH transcription factor 1; HIF1α, hypoxia‐inducible factor 1‐alpha; I/R, ischemic reperfusion; Notch1, neurogenic locus notch homologue protein 1; Numb, NUMB endocytic adaptor protein; W/D, wet‐to‐dry.

To further verify the critical role of miR‐485 suppressed Numb expression in HIF1α alleviated I/R‐induced lung injury in vivo, we transfected the miR‐485 inhibitor or Numb overexpression in vector control or HIF1α overexpression mice. Firstly, mouse models with lung I/R injury in orthotopic mouse lung transplants were established (Figure 7A), after which, W/D ratio and the pathological characteristics of lung tissues were all determined. As shown in Figure 7B, haematoxylin–eosin (HE) staining displayed lower degrees of lung tissue injury in the HIF1α overexpression group (*p* = 0.002), which was reversed in miR‐485 inhibition (*p* = 0.002) or Numb overexpression (*p* = 0.008) groups. In addition, HIF1α overexpression reduced W/D ratio in lung tissues and was increased by miR‐485 inhibition or Numb overexpression groups (Figure 7C, *p* < 0.001), indicating aggravated lung injury. Furthermore, the level of Hes1 expression was increased by HIF1α overexpression, whereas suppressed by miR‐485 inhibition or Numb overexpression (Figure 7D, *p* < 0.001). Collectively, these results suggested that HIF1α alleviated I/R‐induced lung injury and mitophagy via miR‐485 suppressed Numb expression in vivo.

**FIGURE 8:**
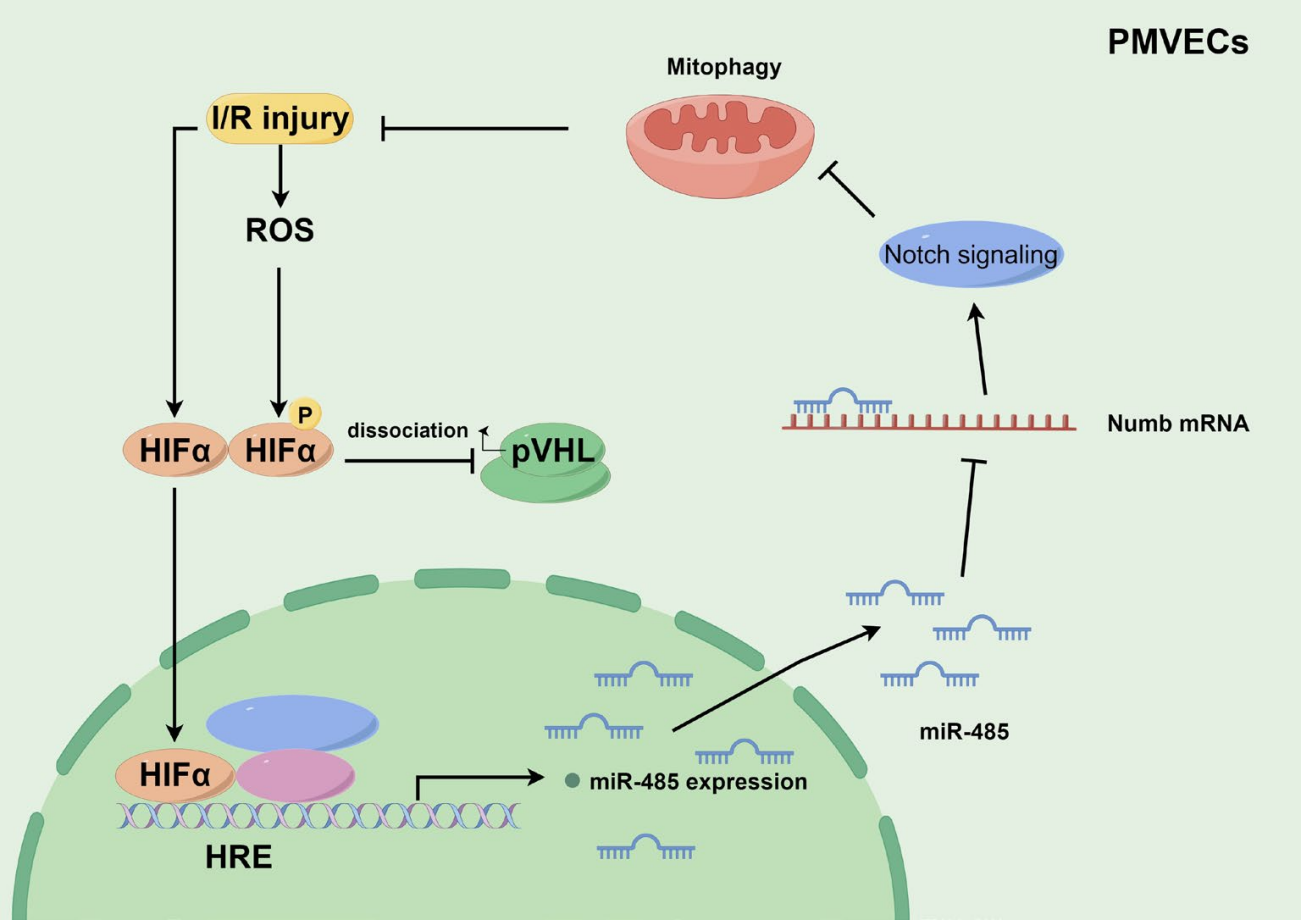
The scientific model diagram showed that HIF1α activates the Notch1 signalling pathway, suppresses mitochondrial dysfunction and excessive mitophagy and transcriptionally activates the expression of miR‐485. The upregulation of miR‐485 subsequently inhibits Numb expression, leading to the activation of Notch signalling and the attenuation of H/R‐induced injury and mitophagy in PMVECs.

In conclusion, our study provides evidence that HIF1α plays a crucial role in protecting PMVECs against H/R‐induced injury and mitophagy. We have demonstrated that HIF1α activates the Notch1 signalling pathway, suppresses mitochondrial dysfunction and excessive mitophagy and transcriptionally activates the expression of miR‐485. The upregulation of miR‐485 subsequently inhibits Numb expression, leading to the activation of Notch signalling and the attenuation of H/R‐induced injury and mitophagy in PMVECs (Figure 8).

## 4 Discussion

Lung IRI remains a significant clinical challenge, particularly in transplantation and critical care medicine [31]. Our study elucidates a novel protective mechanism orchestrated by HIF1α in PMVECs against H/R‐induced injury. We identify a precise signalling axis wherein HIF1α transcriptionally upregulates miR‐485, which in turn targets and represses the negative regulator Numb, thereby amplifying Notch1 signalling to promote cell survival and suppress detrimental mitophagy. This HIF1α–miR‐485–Numb–Notch1 pathway represents a sophisticated adaptive response to ischemic stress.

The central role of HIF1α in cellular adaptation to hypoxia is well‐established [4], but its specific functions in lung IRI are complex and context dependent. While some studies associate HIF1α with pro‐inflammatory responses, a growing body of evidence, corroborated by our data, supports its protective role in endothelial homeostasis during I/R stress [32]. Our findings demonstrate that HIF1α overexpression significantly enhances PMVEC viability and attenuates apoptosis under H/R conditions. This aligns with previous work showing HIF1α‐mediated activation of cytoprotective genes involved in glycolysis, redox balance and apoptosis inhibition [33, 34]. The novelty of our work lies in connecting this HIF1α‐mediated survival directly to the regulation of a specific miRNA and its downstream effector pathways.

A key finding is the demonstration that HIF1α directly binds to the miR‐485 promoter and activates its transcription. While HIF1α is renowned for regulating protein‐coding genes, its role in orchestrating miRNA networks in IRI is less explored. miR‐485 has been implicated in cancer [35, 36] and neuronal apoptosis [24], but its function in endothelial I/R injury was previously unknown. Our results position miR‐485 as a critical downstream effector of HIF1α. The reversal of HIF1α's protective effects by a miR‐485 inhibitor underscores its indispensability in this pathway, suggesting that the beneficial effects of HIF1α stabilisation are largely mediated through this miRNA.

The most significant mechanistic insight from our study is the identification of Numb as a direct target of miR‐485 and the functional consequence of this interaction on Notch1 signalling. Numb is a well‐known endogenous inhibitor of Notch signalling, typically promoting the endocytic degradation of Notch receptors. The Notch pathway plays a dichotomous role in IRI, with its outcomes highly dependent on cellular context and the specific receptors and ligands involved [30]. Our data suggest that in PMVECs under H/R stress, Notch1 activation is protective. We found that HIF1α activation led to upregulated Hes1 expression and promoter activity, indicating Notch1 pathway activation. Crucially, this activation was dependent on miR‐485, as its inhibition suppressed Hes1 despite HIF1α overexpression. The subsequent discovery that miR‐485 directly silences Numb provides an elegant mechanistic link: HIF1α induces miR‐485, which represses the Notch inhibitor Numb, thereby relieving inhibition on the Notch1 pathway and allowing for a pro‐survival signal. This constitutes a novel positive feedback loop within the HIF1α‐Notch axis, significantly advancing our understanding of their crosstalk in endothelial cell fate decisions during stress.

Furthermore, we demonstrate that this axis regulates mitochondrial integrity. H/R‐induced mitochondrial dysfunction and excessive mitophagy are key drivers of IRI [37]. Our results show that HIF1α overexpression promotes a shift towards mitochondrial fusion (increased MFN1) and away from fission (decreased DRP1), concomitantly suppressing mitophagy. This effect was also dependent on the miR‐485‐Numb‐Notch1 axis. This suggests that the pathway's benefits extend beyond pure cell survival to include preservation of cellular energetics and prevention of death signals amplified by damaged mitochondria. The regulation of mitochondrial dynamics by the Notch pathway is an emerging concept, and our work provides a clear mechanistic pathway for this in PMVECs.

The translational potential of our findings is supported by consistent results in our in vivo mouse model of lung I/R injury. The model, involving temporary occlusion of the lung hilum followed by reperfusion, effectively recapitulates key features of clinical IRI, including endothelial barrier dysfunction, inflammation and oxidative stress [38]. The observed attenuation of tissue injury and reduction in the wet‐to‐dry weight ratio (a marker of pulmonary edema) upon HIF1α stabilisation confirms its protective role in a whole‐organ context. Crucially, the abolition of this protection by miR‐485 inhibition or Numb overexpression validates the in vivo significance of the entire axis.

However, the clinical relevance of this model must be interpreted with its limitations in mind. A significant pro of this model is its direct translational relevance to lung transplantation, where unilateral hilar occlusion mimics the intraoperative period of graft implantation. The cons include the inherent simplicity of a model that does not fully capture the complexity of human disease, which often involves comorbid conditions, immune system interactions and pharmacologic interventions. A key limitation of our current in vivo study is the absence of direct lung function measurements, such as arterial blood gas analysis or pulmonary compliance. Incorporating these physiologic parameters in future studies would strengthen the clinical correlation by directly linking molecular mechanisms to functional outcomes. Furthermore, the use of global HIF1α overexpression or knockdown models could have systemic effects; employing cell‐specific (e.g., endothelial‐specific) genetic models would help to precisely delineate the cell‐autonomous effects of this pathway in PMVECs.

In conclusion, our study delineates a novel HIF1α–miR‐485–Numb–Notch1 signalling axis that is crucial for mitigating PMVEC injury and dysregulated mitophagy during H/R stress. By identifying miR‐485 as a key transcriptional target of HIF1α and Numb as its functional effector, we provide a mechanistic explanation for the cross‐talk between hypoxia sensing and cell fate signalling. Targeting this axis, particularly by inhibiting Numb or mimicking miR‐485, presents a promising therapeutic strategy for preventing or treating lung I/R injury. Future research should focus on developing delivery mechanisms for these molecular targets and validating their efficacy in more complex, clinically relevant models.

## Author Contributions

**Shaohua Dai:** conceptualization (equal), formal analysis (equal), investigation (equal), methodology (equal), validation (equal), visualization (equal), writing – original draft (equal). **Xuemei Wan:** conceptualization (equal), supervision (equal), visualization (equal), writing – review and editing (equal). **Lingchun Xia:** conceptualization (equal), resources (equal), software (equal), validation (equal), visualization (equal). **Lei Xu:** conceptualization (equal), data curation (equal), formal analysis (equal), investigation (equal), methodology (equal). **Chunfan Xie:** conceptualization (equal), formal analysis (equal), methodology (equal), visualization (equal). **Guohui Wang:** data curation (equal), supervision (equal), validation (equal), visualization (equal). **Jian Tang:** funding acquisition (equal), investigation (equal), project administration (equal), supervision (equal), writing – review and editing (equal).

## Funding

This work was supported by grants from the National Natural Science Foundation of China (No. 82170109) and The First Affiliated Hospital of Nanchang University Outstanding and Excellent Youth Cultivation Project (No. JQ202103).

## Ethics Statement

All animals were maintained in a pathogen‐free environment and used according to the approved protocols of the Animal Care and Control Committee of The First Affiliated Hospital, Nanchang University and NIH guidelines.

## Consent

The authors have nothing to report.

## Conflicts of Interest

The authors declare no conflicts of interest.

## Data Availability

All data obtained in this study is available from the corresponding author upon request.
